# Providers' and Survivors' Perspectives on Affordability Challenges for Gastrointestinal Cancer Treatment in Two Low Socioeconomic Status States of the Southern United States

**DOI:** 10.1002/cam4.71105

**Published:** 2025-07-30

**Authors:** Maria Pisu, Nataliya V. Ivankova, Jessica Morgan, Courtney P. Williams, Nathan C. English, Burkely P. Smith, Bayley A. Jones, Wendelyn M. Oslock, Yu‐Mei Schoenberger, Ivan I. Herbey, Daniel I. Chu

**Affiliations:** ^1^ Division of General Internal Medicine and Population Science University of Alabama at Birmingham Birmingham Alabama USA; ^2^ O'Neal Comprehensive Cancer Center University of Alabama at Birmingham Birmingham Alabama USA; ^3^ Department of Health Services Administration University of Alabama at Birmingham Birmingham Alabama USA; ^4^ Department of Sociology University of Arkansas Fayetteville Arkansas USA; ^5^ Department of General Surgery University of Cape Town Cape Town South Africa; ^6^ Department of Surgery University of Alabama at Birmingham Birmingham Alabama USA; ^7^ Department of Quality Birmingham Veterans Affairs Medical Center Birmingham Alabama USA

**Keywords:** access to care, affordability, disparities, gastrointestinal cancer, health insurance, poverty

## Abstract

**Introduction:**

Affordability is an access to care domain contributing to disparities in gastrointestinal (GI) cancer outcomes and care, including surgical care. Affordability challenges for GI cancer care in socioeconomically disadvantaged and diverse states of the southern United States are unknown: this paper addresses this knowledge gap.

**Methods:**

We conducted semi‐structured interviews with 32 providers (13 surgeons and 19 other) and 36 survivors (53% colorectal, 19% pancreatic, and 28% esophageal cancer) in Alabama and Mississippi. Questions were about barriers and facilitators to surgical care along five domains, including affordability, that is, the relationship between health care costs, patients' income, health insurance coverage, and the resulting ability to afford care. Verbatim transcripts were analyzed using thematic and content analysis.

**Results:**

Themes were about: (1) affordability‐related underlying context, that is, (i) patients' limited means and competing basic needs priorities, (ii) scarcity of quality medical services, and (iii) rural hospitals' limited means; (2) barriers to medical decision‐making, that is, (i) guideline‐concordant care unfeasible due to poverty and (ii) insurance authorizations and coverage delaying and making care costlier; (3) economic burdens from (i) many types of needed expenses and (ii) billing; and (4) strategies to improve affordability, that is, (i) care adjustments to reduce patients' costs, (ii) community organizations' support, and (iii) burdensome access to resources.

**Conclusions:**

Underlying poverty, scarce quality medical services, and restrictive insurance provisions significantly impact medical decision‐making, access to quality and prompt care, and economic and administrative burdens. Future research should quantify the extent of these challenges and identify programs and policies to address them.

## Abstract 2

This study demonstrates the significant role of affordability‐related challenges in gastrointestinal cancer care, including surgical care, in under‐resourced US states, shedding light on contexts and mechanisms behind existing cancer care and outcome disparities.

## 1 Introduction

Gastrointestinal (GI) cancers, including esophageal, pancreatic, and colorectal cancers, represent approximately 18% of all cancer cases, yet account for up to 28% of all cancer‐related deaths in the United States [1]. This burden is disproportionate in some populations; for example, Black patients or patients in the southern United States experience higher GI cancer mortality compared with their counterparts [2, 3, 4]. Unequal access to guideline‐concordant care may be contributing to this disparity [2, 5]. One example is access to recommended GI cancer surgery, with Black patients less likely to undergo surgery [6, 7, 8, 9], and, if undergoing surgery, more likely to have inadequate resection margins and fewer regional lymphadenectomies [6]. A better understanding of specific barriers to accessing quality GI cancer surgery is warranted, particularly barriers that exist in southern United States which are home to a large share of the US Black population [10].

Barriers to access to medical care can fall under multiple domains, one of which is affordability, that is, the relationship between health care costs, patients' income, health insurance coverage, and the resulting ability to afford medical care [11]. Affordability barriers are salient for cancer patients who may delay or forgo cancer treatment and other aspects of care due to cost [12, 13, 14, 15]. US oncologists identified lack of financial security, including lack of insurance, as the greatest barrier for their patients [16]. Affordability barriers are documented for Black cancer patients who are more likely to limit or forgo care due to cost, have problems with medical bills, and experience financial hardship and health insurance denial after cancer diagnosis [17, 18, 19]. These barriers are salient for the southern United States, which is characterized by high rates of poverty and lower health care quality [20, 21, 22]: In this region, survivors are more likely to forego physician visits due to costs than counterparts in other US regions [23]. Currently, affordability barriers that exist in southern states for GI cancer care, and surgical care in particular, are not clear.

The purpose of this study was to address this knowledge gap by reporting perspectives on affordability barriers of GI cancer care providers and Black and White survivors in two southern US states, Alabama and Mississippi. We report data from a mixed methods research project called Advancing Surgical Cancer Care and Equity in the Deep South (ASCENDS). The goal of ASCENDS was to investigate racial surgical care disparities for GI cancers in Alabama and Mississippi, and to elucidate mechanisms of such disparities, focusing on access to care domains including affordability [11, 24]. We previously reported on themes related to availability and accessibility of GI cancer surgery [25]; here, we focus on themes related to affordability. This work contributes to understanding the health care context faced by patients in the southern United States, and thus by a large share of Black GI cancer patients.

## 2 Materials and Methods

### 2.1 Study Design

The ASCENDS project collected qualitative data from GI cancer providers and survivors through semi‐structured interviews, and quantitative data from cancer survivors through telephone surveys and medical chart abstraction. Here, we report partial results from analyses of semi‐structured interviews. The project was approved by the University of Alabama at Birmingham (UAB) Institutional Review Board (IRB‐300005475). Participants provided informed consent and received a monetary incentive.

### 2.2 Participants and Setting

Health care providers were adults (18 years or older) involved in the care of GI cancer patients, including surgeons, oncologists, nurses, and patient navigators. Providers were identified through internet searches of all Alabama and Mississippi cancer providers, personal contacts of one author (D.I.C.) and colleagues, and ASCENDS survivor participants who were asked to identify the providers or clinics in which they received care. Providers were contacted via email or phone. Using a purposeful snowballing sampling approach, interviewees were asked to identify and recommend members of their teams or other colleagues who may be eligible for an interview.

**FIGURE 1:**
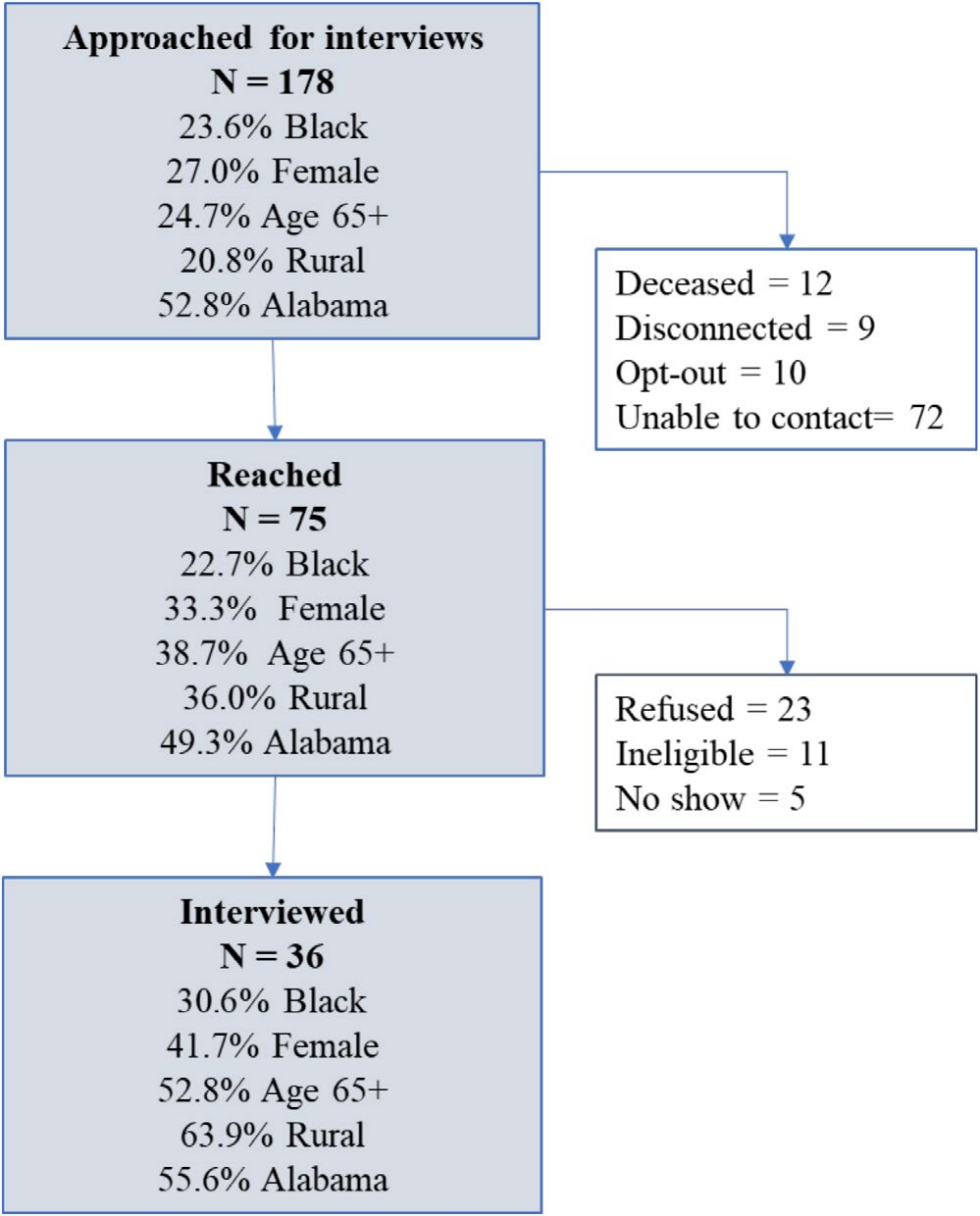
Description of recruitment process and of participants who were contacted for interviews, reached over the phone, and who completed the interviews.

Eligible survivors included adults (18 years or older) who were diagnosed with Stages I–III GI cancer in the previous 5 years, were English‐speaking, and were able to participate in a 1‐h interview. Survivors were identified from hospital registries and from the Alabama Statewide Cancer Registry, and were first contacted by letter (describing study's purpose and providing contact information for opting out), and then by phone. We also partnered with other institutions in Alabama and Mississippi whose personnel contacted survivors via mail, phone, or in person before releasing their contact information. Recruitment was conducted by staff of the O'Neal Comprehensive Cancer Center Participant Recruitment and Assessment shared facility, which assists investigators with recruitment for clinical and behavioral research. We purposefully aimed to recruit a sample of diverse participants, especially survivors who were Black and/or from rural areas (Figure 1).

### 2.3 Data Collection

Interview guides were developed based on the surgical disparities Torain et al.'s framework [26] and the access to care framework by Thomas and Penchansky [11]. Questions focused on barriers and facilitators during the preoperative, interoperative, and postoperative time periods with probes further exploring challenges along the affordability and other access to care domains (Supporting Information). Probes in the providers' interview guide were modified for the specific role of the interviewees, for example, surgeons versus navigators. Two surgical residents and one nurse reviewed and provided feedback on this guide. For survivors' interviews, additional questions asked about problems with medical bills and costs of care. For survivors who did not undergo a surgical procedure, questions focused on their experiences pretreatment, during treatment, and posttreatment. The survivors' interview guide was reviewed with two Black members of our community advisory board (a survivor and a community representative with patient navigation experience) and modified to ensure questions were understandable, relevant, and captured the patient experience during the surgical process.

Interviews with providers were conducted over Zoom by one author (M.P.), a health economist not involved in the practice of clinical GI cancer care. Interviews with survivors were conducted over the phone by a trained interviewer who received study‐specific training from two authors (M.P. and N.V.I.).

### 2.4 Analysis

Interviews were recorded and transcribed verbatim by an independent commercial transcription company. Transcripts, verified by carefully checking the accuracy of transcription, were analyzed using inductive thematic analysis and content analysis [27, 28]. Several qualitative researchers (N.V.I., I.I.H., and B.P.S.) independently analyzed transcripts and regularly met to discuss the emergent codes and themes to establish inter‐coder agreement. A constant comparative method was employed to guide the inductive analytical process to ensure saturation in the data was achieved [29]. To facilitate the analysis, we used NVivo 12.6 Plus, a multifunction software system for qualitative data analysis (Lumivero). Inter‐coder agreement was established at the recommended 90%. We then performed content analysis on the generated themes and subthemes using the counts of text references in NVivo to methodically signify consistencies and differences in opinions within and across different participant groups. For this paper, we selected themes and quotes related to affordability, that is, to health care related costs, patients' income and/or poverty, health insurance coverage and processes, and use of resources to improve affordability.

## 3 Results

Providers (*N* = 32) included surgeons (*n* = 13), medical oncologists (*n* = 2), radiation oncologists (*n* = 2), primary care providers (*n* = 1), nurses (*n* = 6), and patient navigators (*n* = 5). More than two‐thirds (68.8%) practiced in urban areas (Table 1). Survivors (*N* = 36) were on average 64.4 years old, 41.7% female, 66.7% white, and 30.6% Black. The majority (52.8%) had colorectal cancer, followed by esophageal (27.8%) and pancreatic cancer (19.4%). Five survivors (13.9%) did not undergo surgery as part of their treatment.

**TABLE 1:** Characteristics of gastrointestinal cancer survivors and health care providers who participated in interviews.

Variable	Providers (*N* = 32)	Survivors (*N* = 36)
Gender, *n* (%)		
Male	18 (56.3)	21 (58.3)
Female	14 (43.8)	15 (41.7)
Race, *n* (%)		
White	19 (59.4)	24 (66.7)
Black	3 (9.4)	11 (30.6)
Other	8 (25)	1 (2.8)
Missing	2 (6.3)	N/A
Age (years)		
Mean	42.8	64.4
Range	24.6–74.1	44.9–87.0
Cancer type,^a^ *n* (%)		
Colorectal	7 (21.9)	19 (52.8)
Esophageal	6 (18.8)	10 (27.8)
Pancreatic	7 (21.9)	7 (19.4)
Combination	11 (34.4)	N/A
Other	1 (3.1)	N/A
Underwent surgery, *n* (%)		
Yes	N/A	31 (86.1)
No	N/A	5 (13.9)
Urban/rural status, *n* (%)^b^		
Urban	22 (68.8)	11 (30.5)
Rural	8 (25)	23 (63.9)
Missing/other	2 (6.3)	2 (5.6)
State, *n* (%)		
Alabama	25 (78.1)	20 (55.6)
Mississippi	7 (21.9)	16 (44.4)

^a^ Diagnosed cancer (survivors) or cancer of patients cared for (providers).

^b^ Residence (survivors) or setting of hospital or clinic (providers).

Themes and illustrative quotes are reported in Table 2. Four sets of affordability‐related themes emerged as follows: (1) underlying context; (2) barriers to medical decision‐making; (3) economic burdens; and (4) strategies to improve affordability.

**TABLE 2:** Illustrative quotations from providers' and survivors' interviews.

Sets	Themes	Quotes
Affordability underlying context	Patients' limited means and competing basic needs' priorities	“… **you've got a limited amount of money, probably the last thing you want to spend it on is healthcare**. You'd rather spend it on, of course, a roof over your head, food and water on your table…” Surgeon
		“I think there's like a couple of not terrible bills. But right now I couldn't. I'd have to **rob Peter to pay Paul**. So, right now I'm just laying them aside. I will get around to them, but I just really don't have as much money. And whenever that money's gone, it's gone. And that's the way it is here.” White Female, 69, Colorectal Cancer
	Scarcity of quality medical services (providers only)	“So, like **if the outside imaging studies are not of adequate quality, then that may have to be repeated** and therefore, let's say this patient is a surgical candidate upfront, so we're going to really get kind of a full picture of the extent of their eligibility for surgery until we get a quality study; and really, almost the same thing for chemotherapy because with pancreatic cancer, their standard of care is chemotherapy and surgery, and including up to radiation therapy sometimes.” Nurse
	Rural hospitals' limited means (providers only)	“…Not the best equipment. I don't really feel comfortable you know, I don't want to do that, right? We just don't have those—that equipment here. And so that's one of the other factors I would say is important in making sure that somebody has quality outcome.” Surgeon
		“The reason is that staffing in a rural setting is actually difficult for nurses. So there's a nationwide nursing shortage. It is less pronounced in urban areas. It is way more pronounced in rural areas. Because the pickings are slim. And then you have hospitals who are struggling, who are unable to match sort of the salary requirements of other places.” Surgeon
Affordability barriers to medical decision‐making	Poverty making guideline‐concordant care unfeasible (providers only)	“But I mean the challenges there can be transportation, it can be access to chemotherapy, there may not be an oncologist within 40 or 50 miles of them, maybe more; there may not be an oncologist who will actually give appropriate chemotherapy to them, and so we have to consider all of those factors when sort of determining a treatment plan for that patient. **You can't just say, “This is our playbook, this is we how we do it,” you can't say, “I'm going to have somebody do radiation,” if they got to drive 60 miles every day or five days a week for five weeks to get radiation therapy when they don't have gas money to do that**.” Surgeon
		“It's hard, honestly, because… now, there have been a couple of cases where we're able to find a local physician. Like, for instance, if we're recommending chemotherapy up front, we can find a local physician who will be willing to work with them to go ahead and treat them with chemotherapy; there have been cases where unfortunately, the patient just gets so sick that they end up being admitted to our hospital and then, therefore, they can receive their surgery. So, **it's difficult and honestly, our hands are kind of tied with the additional external community support that we can provide to potentially retain those patients** or help them with their treatment options at UAB just because outside of charity care and Cooper Green, there are not a lot of other resources that can help them with that.” Nurse
	Insurance authorizations and coverage delaying and making care costlier	“… that can be arduous and you just… **you want to get a scan and then it's a week before certain insurances will get a pre‐authorization to get their CT scan**; and you're you had surgery scheduled at five days, but they couldn't get their scan for seven, so you got to push it out a day or so, and then that can get to be an issue.” Surgeon
		“Yeah, so I'm just going through them in my brain right now. There are policy issues that focus on insurance and their ability to come to UAB or to get appropriate testing when they do come to UAB. There can be delays because insurance will prevent us from getting a particular test that we need if they'll say, **“We already got one,” or if we won't approve that, that can oftentimes cause an issue as well**.” Nurse
		“…**so they're not in‐network with us and there's nowhere else to send the patients in the state and they do not provide alternatives. They don't tell us we'll send them to [hospital] or send them to [city] or send them here, they just say we don't cover and they will not do single case agreements either.”** Surgeon
		“And I'm going to tell you, that went on the whole time with the diagnosis. It has been**…the doctor is trying to help me figure out too what they'll cover, what they can write a prescription for, what the insurance company has decided I can take**. Not what the doctor writes. No, no, no, no, no. What they decide. If I was a doctor, I would go nuts in this period of time, because I mean, literally those decisions are out of his hands. Literally.” White Female, 71, Pancreatic Cancer
		I was in the hospital—like I said, the tube feeding procedure was, to me, I was set up failure, because when they discharged me home to use the system on my own, they did not give me flush bags; and knowing that the tube, being the size that it is, will clog. So, I'm complaining about why can't I get flush bags, and I was told that, “You couldn't get them, there's none available; we just can't get them.” **And then I was told that, “They cost a lot, and your insurance is probably not going to cover it. They just cost too much.”** Black Female, 58, Pancreatic Cancer
Economic burdens	Burden from many types of needed expenses	“**Lodging, transportation, supportive care medications during the treatment**. So, many patients require medications for pain or other symptoms—diarrhea—that they may be having; and then the **cost of nutrition is not negligible**, particularly for esophagus cancers, many of those patients may be PEC or J‐tube dependent for some period of time or have fairly significant esophagitis and may require switching to like liquid‐based nutrition because of their dysphasia and odynophagia.” Nurse
		“Understanding we have a lot of patients who they actually run out of home health support very quickly, especially these patients who are uninsured or underinsured. And so **oftentimes by the six or eight weeks that we're taking their ostomy down, they actually can't get ostomy supplies anymore**. And so they're on their last bag and they're trying to make that last bag last for another three weeks or something….” Nurse
		“Yeah, medicines are expensive but…now I have…Medicaid so……that covers that. But the hospital…**hospital bill is off the chain**.” Black Female, 60, Pancreatic Cancer
	Burden from billing (survivors only)	“…**they'll send four bills for the same doctor and all four bills will come at the same day with the same doctor's name on it in separate envelopes. How does that make sense when they get to send one bill for that doctor?** …And to me, that's just insanity.” White Female, 47, Colorectal Cancer
Strategies to improve affordability	Care adjustments to reduce patients' costs	“…we do have some **people tell us they need to wait until a certain time before they can have the surgery because that's after they get paid**. So, limited finances… I guess mostly for the transportation; that seems to be the biggest one for people.” Nurse
		“…**someone will elect to wait till January to have their surgery done so that they meet their deductible for the next year**. Or they want to do it before the end of the year so that the deductible is met. And so, sometimes, there's more of a lag than necessarily I would like, but it's because it's financially a big deal for them.” Surgeon
	Community organizations' support	“So, we actually have several different things. I work with a foundation, that's a Pancreatic Cancer Foundation, a patient care advocacy group and as well as the American Cancer Society; our representatives for both of those focus heavily on trying to reduce the economic impact of coming back and forth from therapy. For example, **the private foundation I was telling you about, they'll do things like they'll not only help get gas money to make the travel back and forth, but if it's the day of your clinic visit, they may also go and pick your kids up from school to help assist with things like that**.” Surgeon
	Burdensome access to resources	“Once that is approved or once they get that grant, those run calendar years, so they have it for a year, that grant, and then we have to reapply for that grant. **Sometimes, there's a little waiting time if there's not a grant open, and that's how the oral chemo part works for us. It can be a lengthy process; it can take three or four weeks for a patient to even start that medication**.” Surgeon
		“You know, you got to make sure all that **mountain of paperwork is turned in at a specific time and date** [charity care]” Black Female, 60, Pancreatic Cancer
		“**You have to get all this information to give to them in order to be eligible; and nine times out of ten, when it boils down, you're not eligible**; you just have to have zero dollars. So, I don't care about going through that; but they did offer—each time you go, they'll ask you about it.” Black Female, 68, Pancreatic Cancer

*Note:* Quotations are presented verbatim. Words that exemplify the theme appear in boldface.

### 3.1 Affordability Underlying Context

This set included three themes: (i) patients' limited means and competing basic needs' priorities, (ii) scarcity of quality medical services, and (iii) rural hospitals' limited means.

#### 3.1.1 Patients' Limited Means and Competing Basic Needs' Priorities

Providers acknowledged patients' limited means and the challenges that they face at presentation to surgery. A surgeon reflected that patients, given their poverty status, may not prioritize paying for health care needs given other needs for food and living expenses, and reflected on how this can impact the extent to which patients get needed care: “…when you've got a limited amount of money, probably the last thing you want to spend it on is healthcare” (Table 2). Survivors also commented on having to balance financial obligations: one noted being at the point that “I'd have to rob Peter to pay Paul” and setting aside medical bills for which she had insufficient funds (Table 2).

#### 3.1.2 Scarcity of Quality Medical Services

One theme emerging exclusively from providers was the added cost directly related to the scarcity of adequate diagnostic and treatment facilities in the community, for example, the cost of repeat testing (Table 2). A nurse also discussed how repeat testing caused delays in care: “There can be delays because insurance will prevent us from getting a particular test that we need if they'll say, “We already got one” …” (Table 2).

#### 3.1.3 Rural Hospitals' Limited Means

Surgeons in rural hospitals commented on how hospitals poor financial standing affected the care they can deliver, for example, due to personnel shortages and suboptimal equipment to provide quality surgeries (Table 2).

### 3.2 Affordability‐Related Barriers to Medical Decision‐Making

This set included two themes: (i) poverty making guideline‐concordant care unfeasible and (ii) insurance authorizations and coverage delaying and making care costlier.

#### 3.2.1 Poverty Makes Guideline‐Concordant Care Unfeasible

A concern highlighted by providers specifically was about the challenges that poverty poses when trying to deliver guideline‐concordant care. One provider expressed: “You can't just say, ‘This is our playbook, this is we how we do it,’ you can't say, ‘I'm going to have somebody do radiation,’ if they got to drive 60 miles every day or five days a week for five weeks to get radiation therapy when they don't have gas money to do that” (Table 2). Providers expressed frustration about their limited ability to deliver evidence‐based care due to lack of financial resources for their patients: one commented that “…it's difficult and honestly, our hands are kind of tied with the additional external community support that we can provide to potentially retain those patients” (Table 2).

#### 3.2.2 Insurance Authorizations and Coverage Delay and Make Care Costlier

Providers discussed insurance regulations and processes that made delivering guideline‐concordant care difficult. One surgeon highlighted how insurance authorizations delay care: “… you want to get a scan and then it's a week before certain insurances will get a pre‐authorization to get their CT scan” (Table 2). Moreover, providers noted challenges related to specialists being out of the insurance networks, particularly when care is concentrated in just one large medical center in the state. One surgeon commented: “… so they're not in‐network with us and there's nowhere else to send the patients in the state and they [insurers] do not provide alternatives. They don't tell us send them to [hospital] or send them to [city] or send them here, they just say we don't cover and they will not do single case agreements either.”

Survivors also commented on how insurance impacted their care during and after surgery. A pancreatic cancer survivor stated: “…the doctor is trying to help me figure out too what they'll cover, what they can write a prescription for, what the insurance company has decided I can take,” (Table 2). A pancreatic cancer survivor mentioned how their postoperative care was impacted by the insurance not covering the necessary flush bags: …And then I was told that, “They cost a lot, and your insurance is probably not going to cover it” (Table 2).

### 3.3 Economic Burdens

Two themes under this set were as follows: (i) burden from many types of needed expenses and (ii) burden from billing.

#### 3.3.1 Burden From Many Types of Needed Expenses

Providers highlighted the many costs patients incur throughout treatment, which were not limited to co‐payments for medical services. For example, one provider mentioned: “Lodging, transportation, supportive care medications during the treatment…” (Table 2). Providers mentioned that patients were unable to cover costs for transportation and supportive medications for pain, diarrhea, and nausea, after already spending money on co‐payments. Moreover, they mentioned non‐negligible costs of nutritional supplements and other feeding equipment, especially for esophageal cancer survivors. Medical supplies were sometimes not affordable like one nurse pointed out: “… oftentimes by the six or eight weeks that we're taking their ostomy down, they actually can't get ostomy supplies anymore. And so, they're on their last bag and they're trying to make that last bag last for another three weeks or something…” (Table 2).

Survivors, although mentioning other costs like those for tube feeding supplies, generally commented on medical bills and insurance costs that were exorbitant, especially for hospital encounters and deductibles (Table 2). One participant disclosed starting a GoFundMe to be able to afford medical expenses.

#### 3.3.2 Burden From Billing

Survivors discussed administrative‐related burden repeatedly highlighting “a confusing billing system.” One survivor mentioned that “…they'll send four bills for the same doctor and all four bills will come at the same day with the same doctor's name on it in separate envelopes. How does that make sense when they get to send one bill for that doctor?” (Table 2). Participants commented that confusing billing increases anxiety related to the costs of care.

### 3.4 Strategies to Improve Affordability

This set included three themes: (i) care adjustments to reduce patients' costs, (ii) community organizations' support, and (iii) burdensome access to resources.

#### 3.4.1 Care Adjustments to Reduce Patients' Costs

Providers discussed how they may tailor care to reduce costs for patients. For example, one nurse noted that some patients with limited finances ask to delay their surgery until after they get paid. Providers discussed postponing surgery so patients would be able to meet their deductible for the next year, or, although rarely, providing chemotherapy in the hospital setting rather than in the outpatient setting, or, in urgent cases, suggesting access to the emergency room which allowed them to care for uninsured patients.

#### 3.4.2 Community Organizations' Support

Providers mentioned some available resources, including community organizations that especially helped with transportation costs. One surgeon gave an example: “… the private foundation I was telling you about, they'll do things like they'll not only help get gas money to make the travel back and forth….” (Table 2).

#### 3.4.3 Burdensome Access to Resources

Providers commented on burdensome processes and requirements to apply for some of the available resources. Burdensome applications could result in treatment delays: “Most of the time, we can finally get them a grant or get help through the manufacturer…It can be a lengthy process; it can take three or four weeks for a patient to even start that medication” (Table 2). Similarly, survivors mentioned “mountain of paperwork [to be] turned in at a specific time and date.” One pancreatic cancer survivor pointed out: “You have to get all this information to give to them in order to be eligible; and nine times out of ten, when it boils down, you're not eligible; you just have to have zero dollars” (Table 2).

## 4 Discussion

This study describes affordability‐related barriers to GI cancer care for populations from socioeconomically deprived and racially diverse states like Alabama and Mississippi. Interviewees described patients presenting at the time of surgery with limited ability to afford all needed health care expenses and not entirely prepared for a successful surgery, providers evaluating recommended versus feasible care given insurance limitations and patients' socioeconomic and medical care contexts, and providers having limited ability to help patients with the scarce and at times burdensome resources available. Overall, these affordability challenges impact whether patients receive prompt and quality treatment, how they allocate their limited resources, and the resulting financial and administrative burden they face.

Affordability challenges we reported were strictly interrelated with other domains of access to care, especially with the domains of availability and accessibility [11]. In Alabama and Mississippi, the availability of community‐based cancer care is limited, and transportation barriers limit accessibility to such care and few specialized treatment centers [25]. These limitations have cost consequences, for example, additional costs of repeating diagnostic tests and procedures, or the high cost of transportation. Moreover, limited availability of in‐network cancer treatment facilities that offer complex GI surgical cancer care within a state further leads to cost‐prohibitive care. Previous literature has extensively reported on how insurance status affects cancer care utilization [13, 30]. Our findings add to a growing body of research highlighting the impact of pre‐authorization processes and out‐of‐network provisions on denials or delays of medical care, and exorbitant costs to patients [31, 32]. Survivors we interviewed were aware of how insurance coverage impacted their care in ways that were discordant with their providers' recommendations. These insurance restrictions could impact patient–provider relations and trust. One study found that pre‐authorization was associated with less trust in insurance companies and in the health care system generally [31]. Further studies should assess the consequences of insurance restrictions in terms of the quality and promptness of treatment received, the relationship with the health care system, and ultimately the cancer mortality burden.

Our findings provide some explanation for the impact of socioeconomic status and poverty on cancer care quality and outcomes reported in previous research [33, 34, 35]. In our interviews, providers honestly admitted how poverty impacted their decisions to deliver guideline‐concordant care. Given these findings, it would be important to investigate the value of expanded access to Medicaid, transportation and lodging programs [36], guaranteed income during cancer treatment [37], telemedicine [38], navigation [39, 40], and other initiatives to address the barriers faced by Black and White cancer populations living in poverty.

Our findings expand existing literature on cancer‐related financial hardship. About half of cancer survivors experience such hardship [41, 42], which in part manifests as “coping” behaviors such as delaying or foregoing treatment, and/or sacrificing other forms of treatment [36, 43, 44, 45]. Our findings illustrate what contributes to financial hardship for GI cancer patients in under‐resourced settings, besides co‐payments for physician visits, hospitalizations, drug treatments, and medical procedures. For example, providers highlighted the burden of costs of transportation or of supportive medications and other medical supplies that are not covered by insurance, yet necessary. Thus, affordability of supportive care and supplies ultimately may impact outcomes of surgery and cancer care in general, and the ability of patients to return to normal activities during and after cancer treatment. While resources may exist to help patients afford co‐payments for cancer drugs, they may not exist to cover supportive medications or medical supplies. Further research should investigate the feasibility of fully covering these supportive needs and transportation costs for patients living with limited means. Some evidence suggests that programs to address transportation barriers for cancer patients may be cost‐saving [36]. Lastly, while the financial hardship literature focuses on how patients cope with high costs of cancer care, our findings describe problem‐focused coping strategies of providers as they adjust what, when, and how to deliver care to reduce patients' economic burdens. Therefore, conceptual frameworks of financial hardship could be expanded to include “coping” strategies of providers.

As few other studies [46, 47], we report on burdensome processes of applying for financial help. One study reports that patients face significant barriers in collecting needed documentation and unnecessary or duplicate steps in the application process [46]. Some of our interviewees even reported giving up on seeking assistance due to the administrative burden. Previous research suggested strategies to alleviate this burden, including providing clear and actionable information and individualized assistance throughout the process [46, 47]. These are strategies worth evaluating to help address affordability challenges of GI cancer patients in Alabama and Mississippi.

Given their high representation in the southern United States and in Alabama and Mississippi, the data presented here contribute to a better understanding of the context in which a large share of US Black GI cancer patients seeks and receives care. Despite more than 30% Black participants, however, we cannot report separately the perspectives and experiences of Black and White participants in these data. We aim to do so in the future with the ASCENDS survey of more than 650 GI cancer survivors, of which 26% were Black.

Our study has limitations to consider. Our findings may not be generalizable to regions outside of the Deep South. Alabama and Mississippi are two of only 10 states that have not expanded Medicaid; thus, findings may not generalize to states that have done so. We only included survivors of three GI cancer types, which limits generalizability to other GI cancers like liver and small intestine cancers. We did not interview non‐English speakers and thus may have missed barriers to access to care unique to this population. Finally, participants were survivors who sought out care and may not fully represent those who did not seek treatment and who may have more affordability limitations.

## 5 Conclusion

This study demonstrates the significant role of patient‐ and health system‐related affordability‐related barriers in GI cancer care for the diverse populations in under‐resourced US states. Interviews emphasized multifaceted social needs and the constraints faced by providers in delivering guideline‐concordant care. Future research should quantify the extent of these challenges in socioeconomically disadvantaged states, especially in those where the Black US population is concentrated, and also examine if these challenges differ for Black and White patients within these contexts. Moreover, research should examine how these affordability challenges contribute to existing GI cancer disparities and the effectiveness of programs to address them.

## Author Contributions

**Maria Pisu:** conceptualization (lead), funding acquisition (lead), investigation (lead), writing – original draft (lead). **Nataliya V. Ivankova:** formal analysis (lead), methodology (lead), supervision (lead), writing – review and editing (equal). **Jessica Morgan:** formal analysis (supporting), visualization (lead), writing – original draft (equal). **Courtney P. Williams:** formal analysis (equal), writing – review and editing (equal). **Nathan C. English:** formal analysis (supporting), writing – review and editing (equal). **Burkely P. Smith:** formal analysis (equal), writing – review and editing (equal). **Bayley A. Jones:** formal analysis (supporting), writing – review and editing (equal). **Wendelyn M. Oslock:** formal analysis (supporting), writing – review and editing (equal). **Yu‐Mei Schoenberger:** formal analysis (supporting), project administration (lead), supervision (equal), writing – review and editing (equal). **Ivan I. Herbey:** data curation (lead), formal analysis (equal), writing – review and editing (supporting). **Daniel I. Chu:** conceptualization (lead), funding acquisition (lead), investigation (equal), supervision (lead), writing – review and editing (equal).

## Conflicts of Interest

The authors declare no conflicts of interest.

## Supporting information


Appendix S1.

## Data Availability

The data that support the findings of this study will be available from the corresponding author upon reasonable request.
