# Supplementary material for: Providers' and Survivors' Perspectives on Affordability Challenges for Gastrointestinal Cancer Treatment in Two Low Socioeconomic Status States of the Southern United States
Source: Cancer Med. 2025 Jul 30;14(15):e71105. doi: 10.1002/cam4.71105 (PMC12308909; doi:10.1002/cam4.71105)
Supplement: Supplementary file 1 — Appendix S1. [file CAM4-14-e71105-s001.docx]

**Supplemental Materials**

| **ASCENDS Providers’ Interview Guide** | |
| --- | --- |
| **Preoperative phase of surgical care** | |
| **Question 1** | **In your experience, what are some of the factors or circumstances that make access to surgery easy or difficult?** |
| Probes | What is a typical scenario for patients with GI cancers?  Will you describe some examples of patients for whom this journey may have been different and reasons why that may be?  You mentioned some circumstances related to the patient. Please tell me more about:   - (if mentioned) Affordability factors *(e.g., mentions of missing work, cost of doctors, tests, or drugs, cost of travel, insurance coverage problems, etc)*   You mentioned some circumstances related to the doctors, hospitals or health system in general. Please tell me more about:   - (if mentioned) Affordability factors *(e.g., mentions of insurance coverage, no funds for intake personnel, incentives and disincentives, etc)* |
| **Perioperative phase of surgical care** | |
| **Question 2** | **Now let’s talk about the day patients have surgery. First describe what successful surgery means from your point of view** |
| Probe | What is most important for you as a provider when seeing a patient through surgery? |
| **Question 3** | **In your opinion, what are some of the factors that may impact whether patients receive quality surgery?** |
| Probes | How well do you think the system prepares patients for quality surgery? |
|  | You mentioned some circumstances related to the patient. Please tell me more about:   - (if mentioned) Affordability factors *(e.g., mentions of missing work, cost of doctors, tests, or drugs, cost of travel, insurance coverage problems, etc)* |
|  | You mentioned some circumstances related to the doctors, hospitals, or health system in general. Please tell me more about: |
|  | - (if mentioned) Affordability factors *(e.g., mentions of insurance coverage, no funds for intake personnel, incentives and disincentives, etc)* |
| **Postoperative phase of surgical care** | |
| **Question 4** | **Now let’s talk about the day after surgery, and when patients leave the hospital. Please describe what quality discharge means:** |
| Probe | What is the most important for you as a provider when following up with a patient after surgery? |
| **Question 5** | **What are the factors that may impact whether patients receive quality discharge and follow-up?** |
| Probe | How well do you think the system prepares patients for discharge and follow-up? |
|  | Will you share examples of patients who may not have received quality discharge and follow-up and what circumstances were at play?  Which of the factors we discussed earlier about the period before surgery and the day of surgery, also affect the quality of discharge and follow-up? |
| **Question 6** | **What may your hospital or practice do, or what needs to change at the health system level, to ensure all patients first have access to surgery, and then have quality surgery and follow-up care?** |
| Probe | What would you consider effective and efficient system to ensure all patients have quality surgery they need? |

| **ASCENDS Interview Guide for Survivors** | |
| --- | --- |
| **Preoperative phase of surgical care** | |
| **Question 1** | **Please tell me about how you were diagnosed with cancer, and the medical care you received after that moment.** |
| Probe | Would you tell me who discussed the treatment with you? What treatments were discussed and what did you receive? |
| **Question 2** | **What thoughts went through your mind at the time after hearing the treatment recommended and that you needed surgery?** |
| Probe | What was your understanding of why the surgery was recommended?  What were your thoughts about what surgery would do for you? |
| **Question 3** | **Would you tell me the reasons why you went to or chose that specific hospital where you had surgery?** |
| Probe | Tell me how important the location of hospitals and surgeons was. |
| **Question 4** | **I would like for you to think about the medical visits you had during the time before surgery. Imagine yourself going into the buildings, the clinic, and the doctors, nurses, and staff you saw or interacted with. What experiences, feelings or interactions stood out for you in a positive or negative way?** |
| Probe | How did you feel during these clinic/ hospital visits when you were there? |
| **Question 5** | **Please tell me how easy or difficult it was to get surgery, and why. For example, think about finding a date, scheduling, or confirming and coordinating surgery.** |
| Probes | What were the specific needs (if any) related to your family, work, or health, that you need to work around? What were the specific needs (if any) that you needed the surgical team to work around? |
|  | Did you have any problems in using electronic scheduling systems or any other web-based technology the hospital may have used for scheduling, confirming appointments or other interactions? |
| **Perioperative phase of surgical care** | |
| **Question 6** | **Would you please describe how you prepared for surgery? Think about the instructions you were given, what you had to do, and who helped you.** |
| Probe | How easy or difficult was it for you to understand the instructions you received to prepare for surgery?  Please tell me about which health care provider or other hospital staff helped you in preparing for surgery and how. |
| **Question 7** | **As you did before, please tell me about your experiences, feelings or interactions that stood out for you in a positive or negative way from your time in hospital.** |
| **Question 8** | **Please describe now what happened in the days after the surgery when you were still in the hospital.** |
| Probe | What experiences stood out for you in a positive or negative way from this time? |
| **Postoperative phase of surgical care** | |
| **Question 9** | **Please tell me about the instructions you were given at discharge, and how easy or difficult it was to understand these instructions.** |
| Probe | What were you told to do once you left the hospital to take care of yourself? |
| **Question 10** | **How easy or difficult was it to follow the instructions you were given for the time after the surgery?** |
| Probe | How easy or difficult was it to see your doctor after surgery |
| **Question 11** | **Please describe how you felt at the follow-up visits you had after you left the hospital? What experiences, feelings or interactions stood our for you in a positive or negative way?** |
| **Question 12** | **How easy or difficult was it to deal with costs or medical bills related to the surgery, from before and after you had the surgery?** |
| Probe | Think about co-pays, travel costs, any other costs to get the care you needed. |
| **Question 13** | **Through the overall surgery time, would you say you received the best care possible in a respectful manner?** |
